# Activation of Toll-like receptor 2 induces B_1_ and B_2_ kinin receptors in human gingival fibroblasts and in mouse gingiva

**DOI:** 10.1038/s41598-018-37777-z

**Published:** 2019-02-27

**Authors:** Pedro P. C. Souza, Pernilla Lundberg, Inger Lundgren, Fernando A. C. Magalhães, Claudio M. Costa-Neto, Ulf H. Lerner

**Affiliations:** 1 Department of Physiology and Pathology, São Paulo State University (UNESP), School of Dentistry, Araraquara, SP Brazil; 2 Department of Molecular Periodontology, Umeå University, Umeå, Sweden; 3 Department of Biochemistry and Immunology, Ribeirão Preto Medical School, University of São Paulo, Ribeirão Preto, SP Brazil; 4 Centre for Bone and Arthritis Research at Department of Internal Medicine and Clinical Nutrition, Institute for Medicine, Sahlgrenska Academy at University of Gothenburg, Gothenburg, Sweden; 5 Present Address: School of Dentistry, Federal University of Goiás (UFG), Goiânia, Brazil

## Abstract

The regulation of the kallikrein-kinin system is an important mechanism controlling vasodilation and promoting inflammation. We aimed to investigate the role of Toll-like receptor 2 (TLR2) in regulating kinin B_1_ and B_2_ receptor expression in human gingival fibroblasts and in mouse gingiva. Both *P. gingivalis* LPS and the synthetic TLR2 agonist Pam_2_CSK_4_ increased kinin receptor transcripts. Silencing of TLR2, but not of TLR4, inhibited the induction of kinin receptor transcripts by both *P. gingivalis* LPS and Pam_2_CSK_4_. Human gingival fibroblasts (HGF) exposed to Pam_2_CSK_4_ increased binding sites for bradykinin (BK, B_2_ receptor agonist) and des-Arg^10^-Lys-bradykinin (DALBK, B_1_ receptor agonist). Pre-treatment of HGF for 24 h with Pam_2_CSK_4_ resulted in increased PGE_2_ release in response to BK and DALBK. The increase of B1 and B2 receptor transcripts by *P. gingivalis* LPS was not blocked by IL-1β neutralizing antibody; TNF-α blocking antibody did not affect B_1_ receptor up-regulation, but partially blocked increase of B_2_ receptor mRNA. Injection of *P. gingivalis* LPS in mouse gingiva induced an increase of B_1_ and B_2_ receptor mRNA. These data show that activation of TLR2 in human gingival fibroblasts as well as in mouse gingival tissue leads to increase of B_1_ and B_2_ receptor mRNA and protein.

## Introduction

Kinins are generated by the release from kininogens through the enzymatic action of kallikreins. Since their discovery, these peptides are well known as pro-inflammatory molecules by increasing vasodilation, vascular permeability and cellular migration^1^. The kinin family is composed of bradykinin (BK) and Lys-bradykinin (Lys-BK), both B_2_ receptor agonists, and des-Arg^9^-bradykinin (DABK) and des-Arg^10^-Lys-bradykinin (DALBK), B_1_ receptor agonists^1^. B_2_ receptors are constitutively expressed in many cell types and are responsible for the classical actions of kinins, while B_1_ receptors are induced under pathological conditions and are mainly involved in inflammatory events^1^. Mechanisms controlling the local actions of the kallikrein-kinin system involve release of kinins but also regulation of their receptors^2^. Thus, pro-inflammatory molecules such as cytokines and lipopolysaccharide (LPS) regulate B_1_ and B_2_ receptor expression^3,4^.

Periodontal disease is a highly prevalent chronic inflammatory disease of the periodontium causing loss of gingival tissue, periodontal ligament and tooth-supporting bone. Colonization of the root surfaces on teeth by complex subgingival biofilms, containing several gram-negative bacteria, including *Porphyromonas gingivalis*, initiates the cascade of a wide variety of events leading to infiltration of inflammatory cells and the production of molecules that can disturb the remodeling of periodontal tissues, eventually leading to loss of alveolar bone and loosened teeth^5^. The presence of *P. gingivalis* impedes or modulates the host protective mechanisms in many different ways and is associated with diseased sites. Therefore, *P. gingivalis* is potentially a keystone pathogen that modifies the environment supporting the bacterial community to promote periodontal disease^6^.

We have reported that kinins may play important roles in periodontitis^7^. Accordingly, B_1_ and B_2_ receptors are expressed on osteoblasts and fibroblasts and activation of these receptors causes enhanced bone resorption mediated by increased prostaglandin E_2_ (PGE_2_) formation in both cell types and enhanced expression of receptor activator of nuclear factor-κB ligand (RANKL) in osteoblasts^3,8,9^. Interestingly, *P. gingivalis* expresses an arginine specific cysteine proteinase (Arg-gingipain-1/RGP-1) that can release kinins from kininogens^10^, facilitated by components of the kallikrein-kinin system binding to gingipains on the cell surface of *P. gingivalis*^11^.

Toll-like receptors are a family of pattern recognition receptors that recognize a plethora of pathogen-associated molecular patterns (PAMPs). To the PAMPs belongs lipopolysaccharide (LPS) from gram-negative bacteria, which is recognized by Toll-like receptors 4 (TLR4)^12^. The importance of TLR4 for periodontal disease is well studied, but much less is known on the role of TLR2. Interestingly, *P. gingivalis* has the capacity to activate both TLR2 and TLR4^13,14^. Recently, we reported that *P. gingivalis* stimulates osteoclast formation *in vitro* and causes inflammation induced bone loss *in vivo* through activation of TLR2^15^. This observation and the fact that periodontitis induced by *P. gingivalis* can not be observed in mice with genetic deletion of TLR2 indicates that TLR2 is also important for the pathogenic properties of *P. gingivalis* in periodontal disease^16–18^.

Data from human and mouse studies have evidenced an association between periodontal disease and rheumatoid arthritis (RA)^19–21^. The observation that alveolar bone loss in periodontitis patients precede the clinical onset of symptoms of RA^21^, together with the fact that treatment of periodontitis seems to reduce the severity of RA^22,23^ indicates a possible cause relationship between the two diseases. Further support for a role of oral infection in RA are studies in mice showing that oral infection with *P. gingivalis* aggravates arthritic bone erosions in collagen-induced arthritis^22,24^. The pathogenetic mechanisms involved were, at least in part, dependent on Th17 cells through the activation of TLR2 by *P. gingivalis*^24^. Further supporting an association between periodontal disease and RA is the observation that DNA from *P. gingivalis* has been detected in serum and synovial fluid from RA patients^25^. The routes used by *P. gingvivalis* to invade blood vessels in the periodontium and to reach the joints through the circulation are still unknown, but may be attributed to local activation in the periodontal tissues of the kallikrein-kinin system. This hypothesis is supported by the fact that local vascular permeability and bacterial spreading can be enhanced by *P. gingivalis* through a mechanism that was inhibited by decreasing kinin activity, either by administration of angiotensin converting enzyme (ACE), acting as a kininase enzyme, or by a kinin B2 receptor antagonist. In contrast, increased kinin activity by administration of BK, or the ACE inhibitor captopril, enhanced vascular permeability and bacterial spreading induced by infection with *P. gingivalis*^26^. Interestingly, the ability of *P. gingivalis* to disseminate was strain specific and correlated to generation of kinin activity. Thus, local regulation of kinin receptors in gingival fibroblasts could contribute by increasing the response to BK, leading to the generation of vasoactive mediators, such as prostaglandins, and by promoting bacterial spreading and aggravation of RA in periodontitis patients. In the present study, we have investigated the role of TLR2 for the local regulation of kinin receptors and report the novel finding that activation of TLR2 directly increases the expression of functional B_1_ and B_2_ receptors in human gingival fibroblasts as well as in mouse gingival tissue.

## Results

### Induction of *BDKRB1 and BDKRB2* mRNA expression by *P. gingivalis* LPS and Pam_2_CSK_4_ in HGF

**Figure 1:**
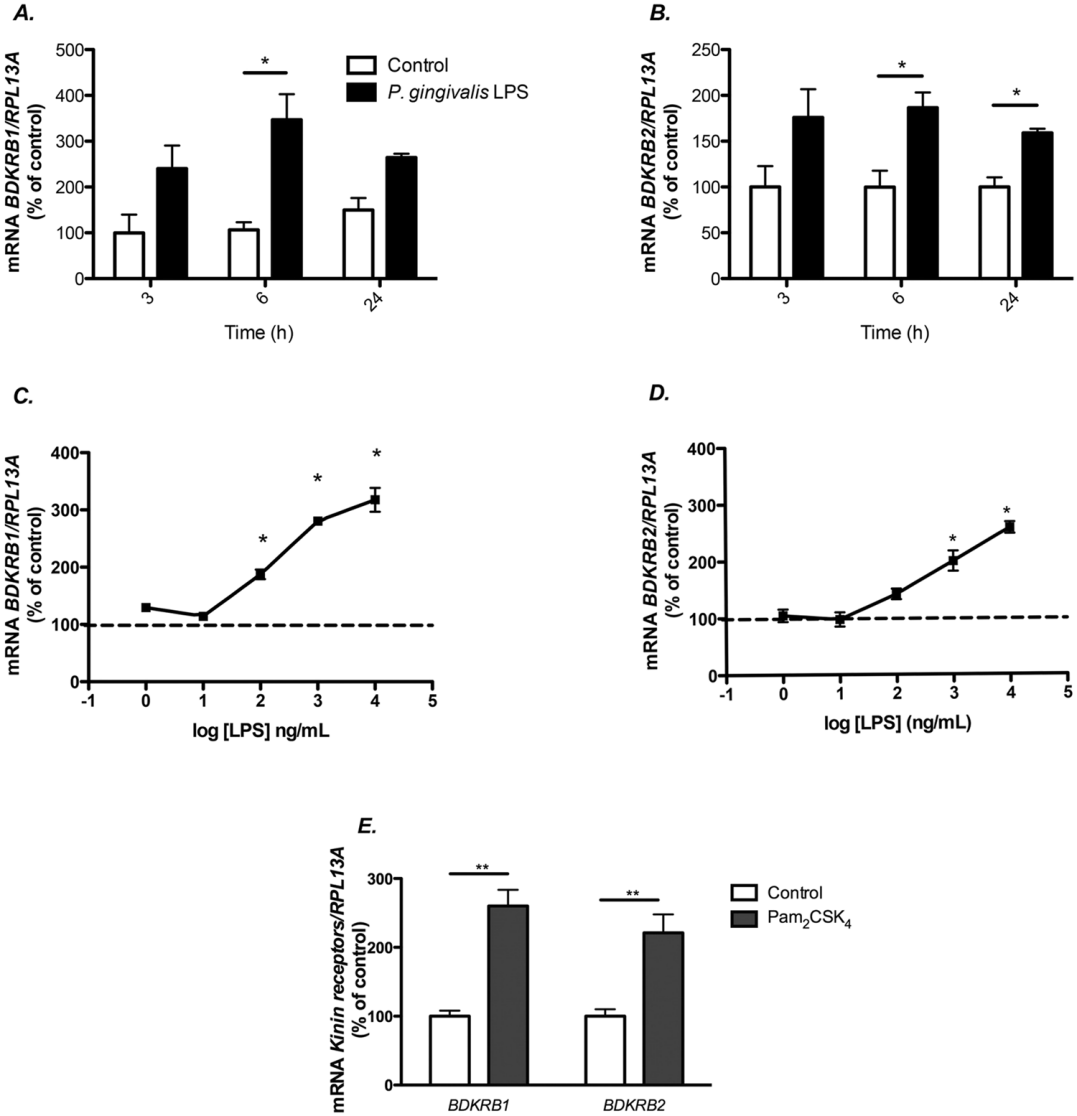
*P. gingivalis* LPS and the TLR2 agonist Pam_2_CSK_4_ increase the expression of *BDKRB1* and *BDKRB2* mRNA in human gingival fibroblasts. Time-course of the expression of *BDKRB1* and *BDKRB2* in human gingival fibroblasts cultured in the presence or absence of 10 μg/mL of *P. gingivalis* LPS (**A**,**B**). *P. gingivalis* LPS dose dependently increased mRNA expression of *BDKRB1* (**C**) and *BDKRB2* (**D**) in human gingival fibroblasts after 6 h of treatment with LPS. Pam_2_CSK_4_ (50 ng/mL) increased *BDKRB1* and *BDKRB2* mRNA in human gingival fibroblasts after 6 h of treatment (**E**). Data were normalized against *RPL13A* and are expressed as percentage of the means for the controls at 3 h (**A**) or controls (**B**–**E**), which was arbitrarily set to 100%. Values represent means for 3 wells/experimental group and SEM is shown as vertical bar. * and ** indicate significant difference to untreated control cells, *P* < 0.05 and *P* < 0.01, respectively. Statistical significance was determined using Student’s t test (*A, B* and *E*) or one-way analysis of variance (ANOVA), with Levene’s homogeneity test and Dunnet’s T3 post hoc test (**C**,**D**).

Human gingival fibroblasts were isolated from an individual without any clinical signs of gingival inflammation. Exposure of these cells to *P. gingivalis* LPS (10 μg/ml) for 3–24 hours resulted in time-dependent increased expression of both *BDKRB1* (Fig. 1A) and *BDKRB2* mRNA (Fig. 1B). The upregulation of *BDKRB1* and *BDKRB2* mRNA caused by *P. gingivalis* LPS was concentration dependent, with stimulatory effects seen at and above 100 ng/ml (Fig. 1C,D). Expression of *IL6* mRNA has previously been reported to be upregulated by *P. gingivalis* LPS^27^; in the present experiments, increased IL6 mRNA was seen at the same concentrations as those stimulating kinin receptor expression (data not shown). *BDKRB1 and BDKRB2* mRNA expression was enhanced also by the synthetic TLR2 agonist Pam_2_CSK_4_ (50 ng/mL) (Fig. 1E).

**Figure 2:**
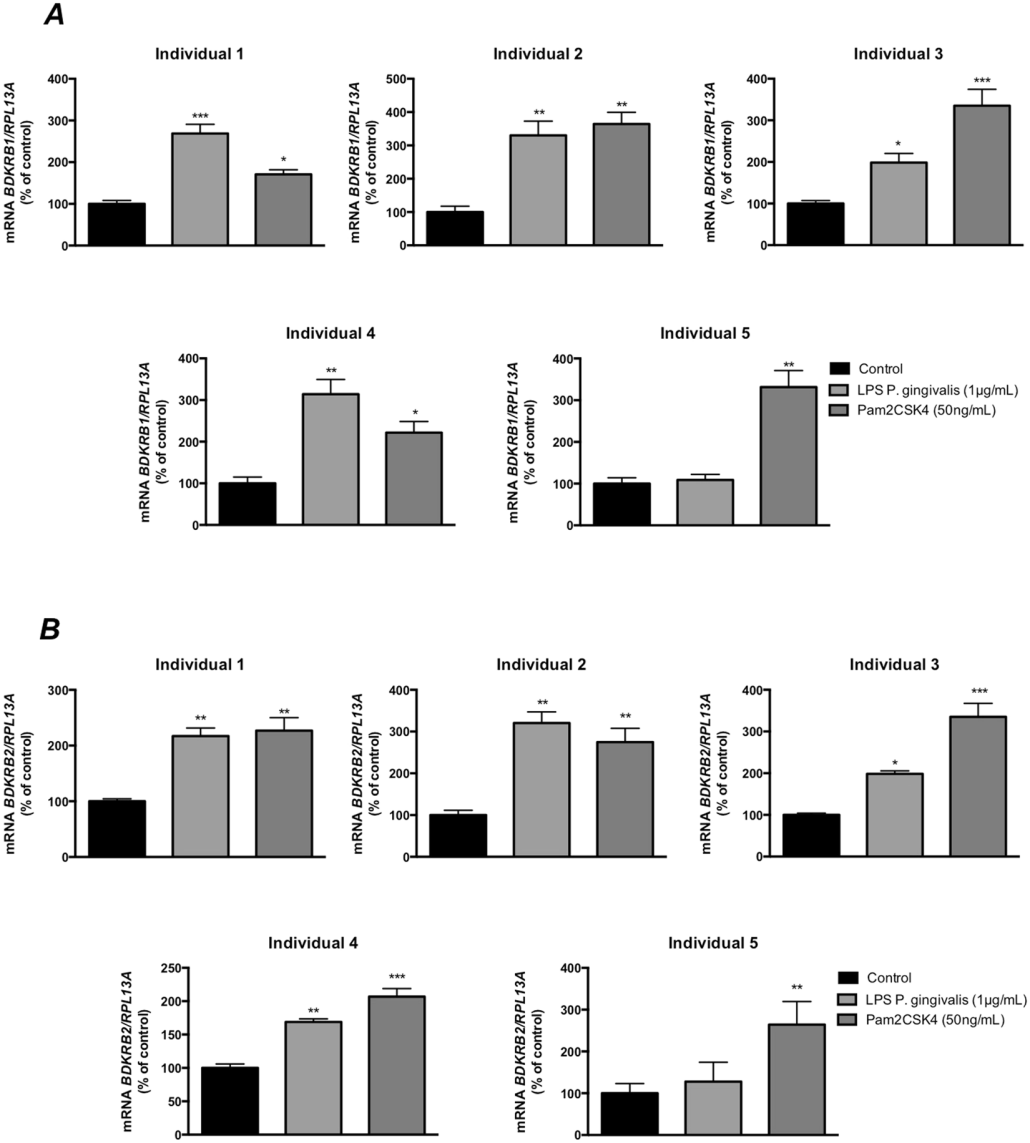
*P. gingivalis* LPS and the TLR2 agonist Pam_2_CSK_4_ increase the expression of B1 and B2 kinin transcripts in human gingival fibroblasts from different individuals. *BDKRB1* (**A**) and *BDKRB2* (**B**) were up-regulated after 6 h of exposure to *P. gingivalis* LPS (1 μg/mL) or Pam_2_CSK_4_ (50 ng/mL) in cells isolated from five individuals. Each bar represents the average of 3 wells/experimental group and SEM is given as vertical bars. *** and *** indicate significant difference to untreated control cells, *P* < 0.05, *P* < 0.01 and *P* < 0.001 respectively. Statistical significance was determined using one-way analysis of variance (ANOVA), with Levene’s homogeneity test and Dunnet’s T3 post hoc test.

In order to evaluate if regulation of kinin receptor expression by *P. gingivalis* LPS and Pam_2_CSK_4_ in gingival fibroblasts was a general phenomenon, we incubated cells isolated from five different individuals with these test substances. In cells from all five individuals, Pam_2_CSK_4_ (50 ng/mL) significantly increased both *BDKRB1 and BDKRB2* mRNA expression (Fig. 2). *P. gingivalis* LPS (1 μg/mL) significantly increased both *BDKRB1 and BDKRB2* mRNA expression in cells from four of the five patients (Fig. 2).

### Induction of *Bdkrb1 and Bdkrb2* mRNA expression by *P. gingivalis* LPS in mouse gingiva

**Figure 3:**
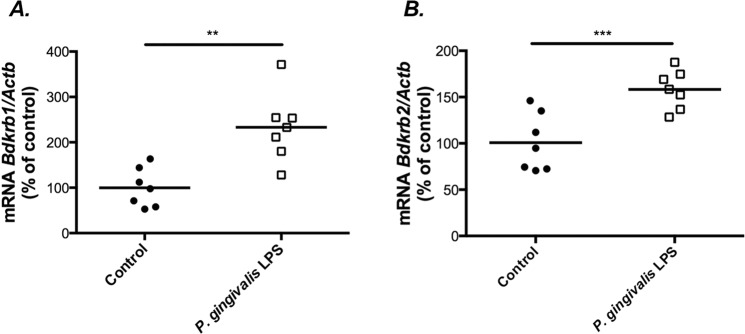
*P. gingivalis* LPS increases the expression of *Bdkrb1* and *Bdkrb2* in mouse gingiva. Injection of LPS from *P. gingivalis* (3 μg) every other day for 14 days increases the expression of *Bdkrb1* (**A**) and *Bdkrb2* (**B**) in mouse gingiva in comparison with injection of vehicle (Control). The expression was analysed using Taqman assays. Data were normalized against *Actb* and are expressed as percent of the means for the controls, which was arbitrarily set to 100%. Each symbol represents data from one mouse. The horizontal line represents the mean for each experimental group. ** and *** indicate significant difference to untreated mice, *P* < 0.01 and *P* < 0.001, respectively. Statistical analysis was determined using Student’s unpaired t-test.

In order to assess if the upregulation of BK receptors by *P. gingivalis* LPS observed in the fibroblasts cultures could be observed also *in vivo*, we locally exposed gingival tissue in mice to the bacterial LPS. Injection of *P. gingivalis* LPS (3 μg) every other day for 14 days in mouse gingiva enhanced the mRNA expression of *Bdkrb1* and *Bdkrb2* (Fig. 3A,B). *Bdkrb1* mRNA was increased by 2.3-fold (Fig. 3A), while *Bdkrb2* mRNA was increased by 1.6-fold (Fig. 3B).

### LPS from *P. gingivalis* and Pam_2_CSK_4_ up-regulate kinin receptor transcripts selectively via TLR2

**Figure 4:**
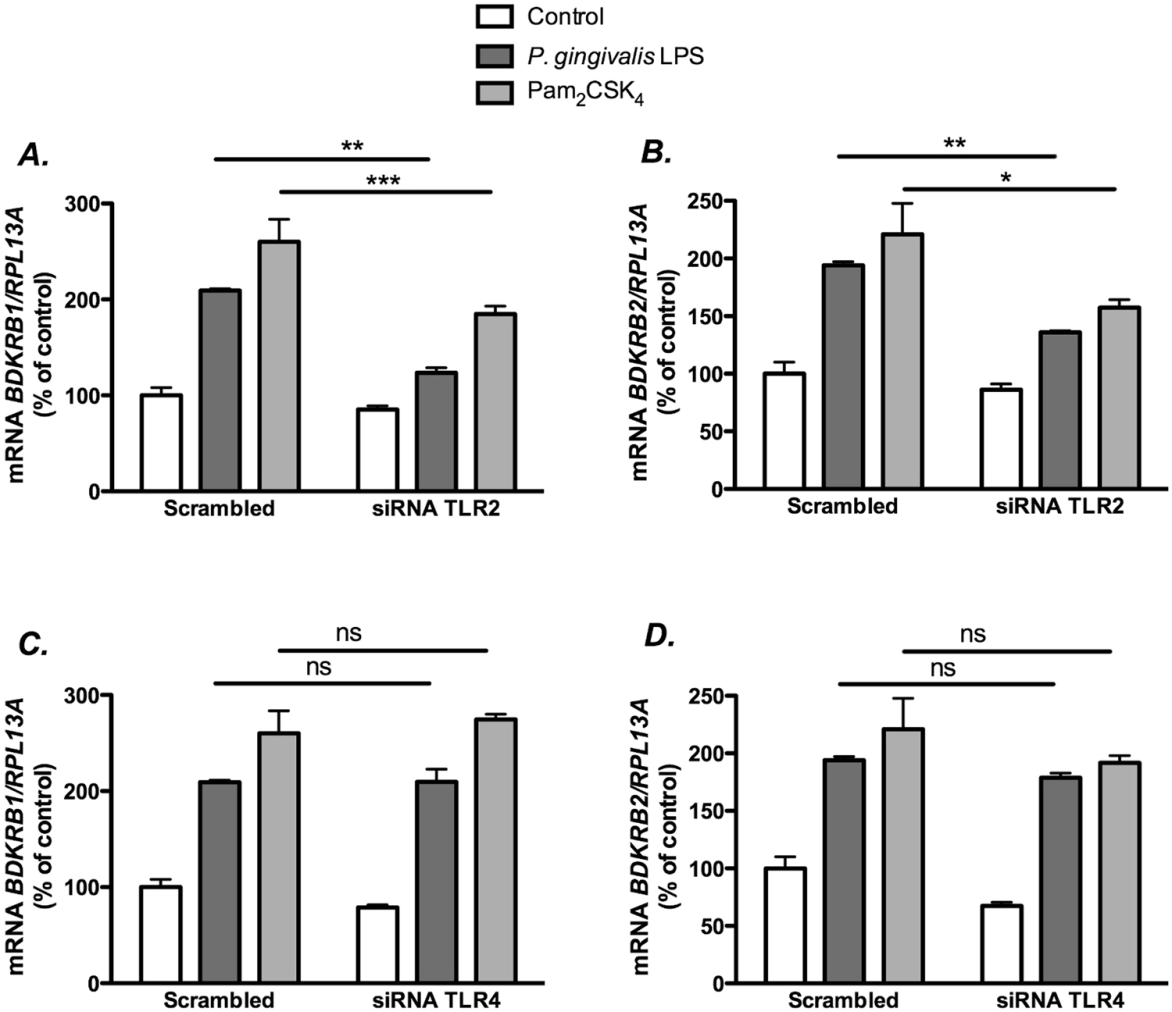
The up-regulation of *BDKRB1* and *BDKRB2* mRNA by LPS from *P. gingivalis* is mediated by TLR2. Gingival fibroblasts were transfected with a scrambled siRNA or siRNA targeting TLR2 (**A**,**B**) or TLR4 (**C**,**D**). Twenty-four hours after transfection, the cells were exposed to LPS form *P. gingivalis* (1 μg/mL) or Pam_2_CSK_4_ (50 ng/mL). After 6 h, the expression of *BDKRB1* (**A**,**C**) and *BDKRB2* (**B**,**D**) mRNA was analyzed by qPCR using Taqman Assays. Data were normalized against *RPL13A* and expressed as percent of control which was arbitrarily set to 100%. Data are expressed as means ± SEM (n = 4 wells/experimental group). *, ** and *** indicate significant difference, *P* < 0.05, *P* < 0.01 and *P* < 0.001, respectively. Statistical analysis was determined using two-way analysis of variance (ANOVA), with Levene’s homogeneity test and Tukey post hoc test. The difference in *P. gingivalis*-induced response with and without silencing analyzed by two-way ANOVA was statistically significant (interaction P value in (**A**) and (**B**) was P < 0.01).

In order to confirm that up-regulation of kinin receptors by the TLR2 receptor agonists used was a specific effect of TLR2 receptor activation, we knocked down TLR2 by using small interfering RNA designed to silence *TLR2* (TLR2-siRNA). To rule out the contribution of TLR4, we also silenced TLR4 using TLR4-siRNA. The mRNA expression levels of *TLR2* and *TLR4* were decreased by 90%, as compared to cells transfected with a control (scrambled) siRNA (SCR-siRNA; data not shown). Our results showed that knockdown of TLR2 significantly decreased the enhancement of *BDKRB1* and *BDKRB2* mRNA induced by *P. gingivalis* LPS, as well as by Pam_2_CSK_4_ (Fig. 4A,B). In contrast, knockdown of *TLR4* did not significantly affect kinin receptor expression induced by *P. gingivalis* LPS or by Pam_2_CSK_4_ (Fig. 4C,D).

### TLR2 agonists up-regulate kinin receptors at protein level

**Figure 5:**
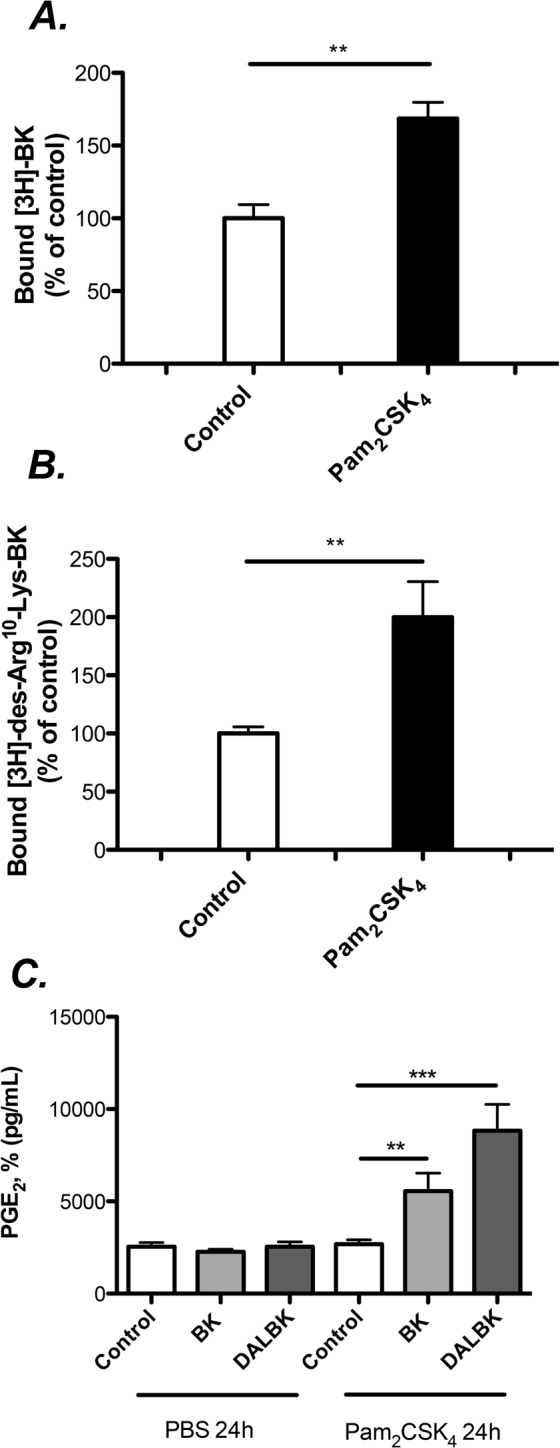
TLR2 activation by Pam_2_CSK_4_ enhances the number of B1 and B2 binding sites, and the prostaglandin response induced by kinins. Gingival fibroblasts were pre-treated with Pam_2_CSK_4_ (50 ng/ml) or PBS (controls) for 24 h (**A**–**C**). The cells were then exposed to radiolabelled ligands for 90 minutes for binding analysis, (**A**,**B**) or treated for additional 24 h with kinins in order to assess the amount of PGE_2_ released (**C**). The results represent means ± SEM of 4 wells/experimental group. ** and *** indicate significant difference, *P* < 0.01 and *P* < 0.001, respectively. Statistical analysis was determined using Student’s unpaired t-test (**A**,**B**) or determined using two-way analysis of variance (ANOVA), with Levene’s homogeneity test and Tukey post hoc test. (**C**) The difference in PGE_2_ release with and without pretreatment with Pam_2_CSK_4_ by two-way ANOVA was statistically significant (interaction P value was P < 0.01).

As shown in Fig. 5A,B, gingival fibroblasts pre-treated with Pam_2_CSK_4_ for 24 h exhibited enhanced binding to [^3^H]-BK and [^3^H]-DALBK, evidencing that the number of correctly folded receptor proteins capable of binding to the kinin receptors was enhanced.

To analyze the functional relevance of the up-regulation of B1 and B2, we took advantage of the fact that activation of both receptors are linked to increased formation of PGE_2_ in gingival fibroblasts^28,29^, as well as in many other cell types. When the fibroblasts were pre-treated with Pam_2_CSK_4_ for 6 h (50 ng/mL), the subsequent PGE_2_ responses to both BK and DALBK were enhanced (Fig. 5C), indicating that activation of TLR2 results in increased number of functional kinin receptors.

### Analysis of the participation of IL-1β and TNF-α in *P. gingivalis* LPS-induced kinin receptor expression

**Figure 6:**
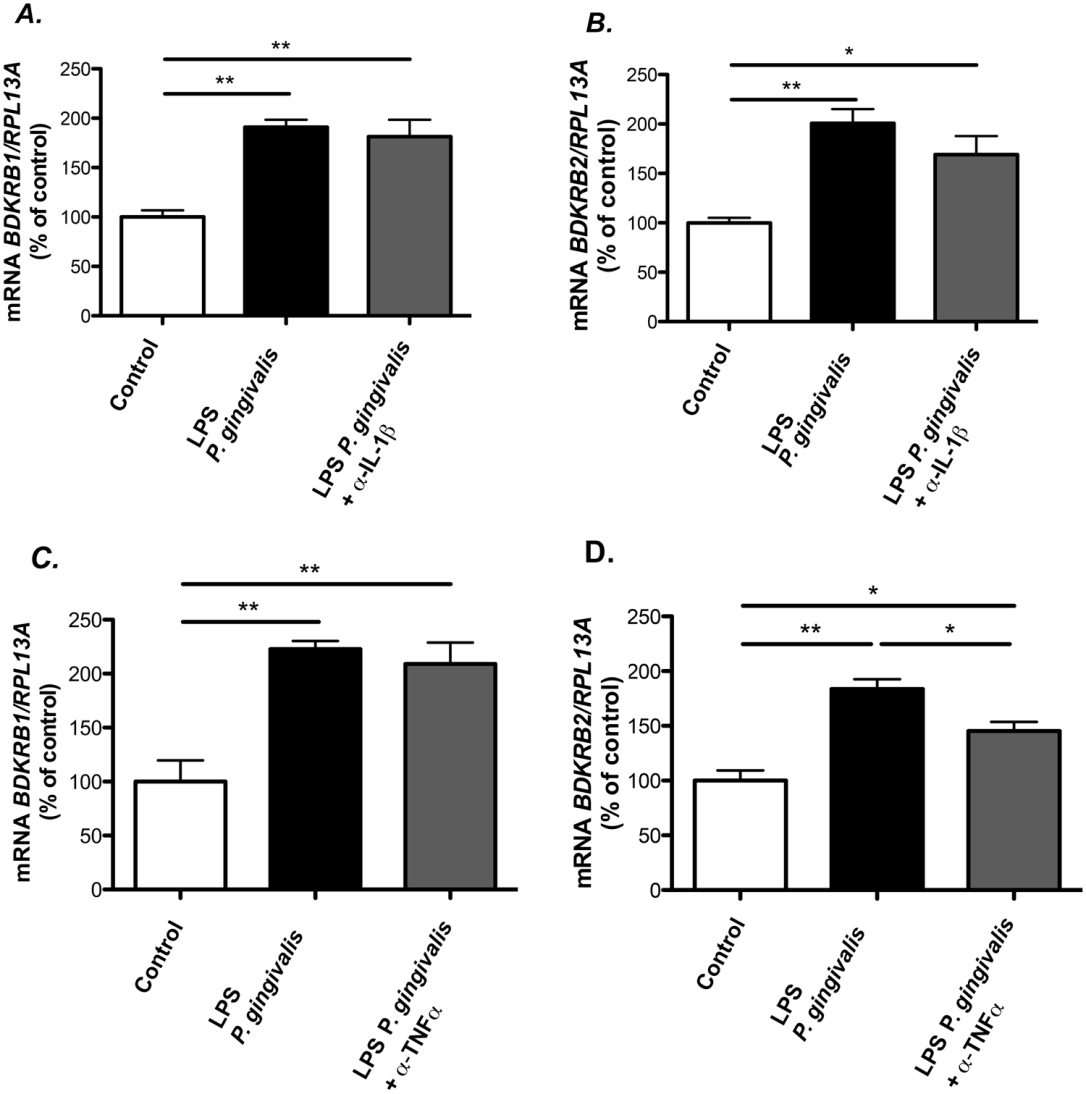
The role of IL-1β and TNF-α in *P. gingivalis* LPS mediated up-regulation of *BDKRB1* and *BDKRB2* mRNA. Gingival fibroblasts were exposed to 1 μg/mL of LPS from *P. gingivalis* for 6 h in the presence or absence of anti-IL-1β (0.3 μg/mL) (**A**,**B**) or anti-TNF-α (1 μg/mL) (**C**,**D**) and the expression of *BDKRB1* (**A**,**C**) and *BDKRB2* (**B**,**D**) mRNA was analyzed by qPCR using Taqman Assays. Data were normalized against *RPL13A* and expressed as percent of control which was arbitrarily set to 100%. Data are expressed as means ± SEM (n = 4 wells/experimental group. *** and *** indicate significant difference, *P* < 0.05, *P* < 0.01 and *P* < 0.001, respectively. Statistical analysis was determined using one-way analysis of variance (ANOVA), with Levene’s homogeneity test and Tukey post hoc test.

In the human gingival fibroblasts, LPS from *P. gingivalis* induced the expression of *IL-1β* and *TNF-α* mRNAs, which were undetectable in control cells not exposed to LPS (data not shown). We, therefore, evaluated if these cytokines could participate in kinin receptor expression induced *P. gingivalis* LPS and for these purpose made use of specific neutralizing antibodies tested to verify their effectiveness (Supplemental 2). Neither the antibody neutralizing IL-1β (Fig. 6A), nor the one neutralizing TNF-α (Fig. 6C), affected *P. gingivalis* LPS (1 μg/mL)-induced increase of the mRNA expression of *BDKRB1*. At variance, although the treatment with the IL-1β neutralizing antibody caused no effect on *BDKRB2* mRNA induced by *P. gingivalis* LPS (Fig. 6B), the TNF-α neutralizing antibody partially inhibited the up-regulation of *BDKRB2* mRNA (Fig. 6D).

## Discussion

In the present study, we report that the mRNAs encoding for the B_1_ and B_2_ kinin receptors are among those genes regulated by LPS from the periodontopathogenic bacterium *P. gingivalis*, both *in vitro* in human gingival fibroblasts and *in vivo* in mouse gingiva. Interestingly, it has been demonstrated that expression of TLR2 mRNA and protein, one of the receptors activated by *P. gingivalis* is enhanced by activation of B2 kinin receptor, indicating a bidirectional regulation of kinin receptors and TLR2 by their cognate ligands^30^.

In order to escape from the host recognition by the innate immune system and promote its adaptive fitness in the mammalian host, *P. gingivalis* LPS may elicit different responses when bound to TLR2 or TLR4^13,14^. The heterogeneous responses of *P. gingivalis* LPS observed *in vitro* and *in vivo* may be due to the fact that many preparations are contaminated with lipoproteins or other lipid species^31,32^. Although TLRs are mainly present in inflammatory cells, it has been shown that gingival fibroblasts express a number of proteins belonging to the TLR family, including TLR2 and TLR4^33^. Such data suggest that resident gingival fibroblasts might participate in the first recognition of pathogens. In line with this observation, we report here that *P. gingivalis* LPS was able to upregulate B_1_ and B_2_ receptors, both at the mRNA and at the protein levels. Up-regulation of B_1_ and B_2_ receptors in an organ culture model of tracheal segments isolated from mice by *Salmonella* LPS (TLR2 and TLR4 agonist) and by polyinosinic polycytidylic acid (TLR3 agonist) has also been reported^34^. Here, we show that the induction of B_1_ and B_2_ receptor expression by *P. gingivalis* LPS is an effect directly mediated by TLR2. One evidence for this is that Pam_2_CSK_4_, a specific TLR2 synthetic agonist, also upregulated B_1_ and B_2_ receptors in the human gingival fibroblasts. Furthermore, we also demonstrated that the effects elicited by *P. gingivalis* LPS and Pam_2_CSK_4_ were decreased in gingival fibroblasts in which TLR2 expression was robustly decreased by siRNA silencing, but remained unchanged in cells in which TLR4 was likewise siRNA-silenced, although we can not exclude that some remaining TLR4 protein after silencing might have contributed to the response.

It was previously reported that *E. coli* LPS is capable of regulating the expression of *Bdkrb1* in a mouse paw edema model^35^. In this multicellular system, the purposed sequence of events that leads to the up-regulation of B_1_ receptor by *E. coli* involves the release of pro-inflammatory cytokines such as IL-1β and TNF-α, the release of chemoattractant molecules and neutrophil influx. Some years later, it was shown that LPS from *P. gingivalis* also up-regulates *Bdkrb1* in the same model, by a mechanism that also involves neutrophil influx and TNF-α production^4^. Noteworthy, in the present study we show that human gingival fibroblasts are capable of up-regulating both *BDKRB1* and *BDKRB2* independently of IL-1β and in the case of BDKRB2, partially dependent of TNF-α.

We have previously reported that IL-1β and TNF-α enhance the expression of *BDKRB1* and *BDKRB2* in human gingival fibroblasts^3^. Since induction of pro-inflammatory cytokines is a well-recognized response to TLR2 activation^36^, we investigated if these cytokines mediated the effects by *P. gingivalis* LPS and Pam_2_CSK_4_ on kinin receptor expression. Using antibodies that specifically neutralize the effects of IL-1β and TNF-α, we show that up-regulation of *BDKRB1* occurs independently of the production of both cytokines, whereas *BDKRB2* up-regulation is partially dependent on TNF-α production but independent on IL-1β. In the mouse paw model, where inflammatory cells can be recruited to the inflamed site, neutrophil influx and TNF-α production are important events for the regulation of *BDKRB1* levels by *P. gingivalis* LPS^4^. Although TNF-α expressing neutrophils are present in the inflamed gingiva during periodontitis^37^, the up-regulation of *BDKRB1* in gingival fibroblasts, independently of IL-1β and TNF-α, may be of importance for the actions of kinins in the periodontium in chronic inflammation. As regards the *BDKRB2* up-regulation, it has previously been shown that the up-regulation of this receptor by cardiac myocytes challenged with LPS was partially dependent on TNF-α production^38^, in agreement with our data. Nevertheless, it is interesting to note that the *BDKRB2* up-regulation by *P. gingivalis* LPS was not completely inhibited by TNF-α neutralizing antibody in the gingival fibroblasts, which means that other TNF-α-independent pathways may also be involved in the regulation of the expression of this receptor.

**Figure 7:**
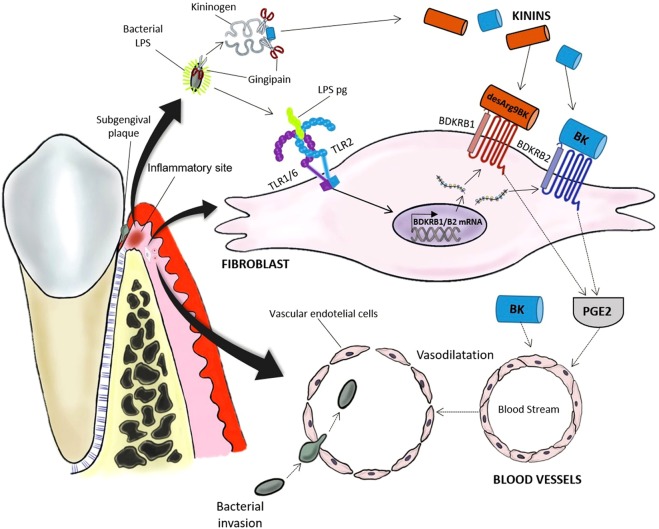
Proposed role of kinin receptors in gingival fibroblasts for the invasion of *P. gingivalis* in gingival blood vessels. LPS from *P. gingivalis* is released from the biofilm on teeth at the inflammatory site and binds to TLR2, composed either by the heterodimer TLR1/TLR2 or TLR6/TLR2 in the cell membrane of human gingival fibroblasts. At the same time, the kinin-releasing protease gingipain expressed by *P. gingivalis* promotes the generation of kinins at the inflammatory site. Activation of TLR2 leads to the expression of kinin receptor mRNA and protein by gingival fibroblasts. The binding of BK and DALBK to B2 and B1 receptors, respectively, expressed by the fibroblasts leads to the release of PGE_2_. Kinins and PGE2 may act as vasodilator agents, facilitating the penetration of bacteria into the blood vessels and their spreading to other tissues.

The data presented here may be of clinical relevance, since activation of TLR2 by *P. gingivalis* is associated with the aggravation of experimental arthritis in mice^24^. In humans, an association between periodontitis and RA has been demonstrated^21,22,24^. One possible mechanism has been suggested to be due to the presence of PAMPs derived from oral bacteria in the diseased joints. Supporting this view, DNA from periodontopathogenic bacteria can be detected in the serum and synovial fluid from patients with RA and psoriatic arthritis^25,39^. The mechanism underlying the invasion of periodontogenic bacteria is still elusive, but one possible route could be local activation of the kallikrein-kinin system from a sequestered infection site to promote vasodilation and facilitate invasion^26^. Our data can be reconciled with this hypothesis, since activation of TLR2 by *P. gingivalis* LPS not only increased B1 and B2 receptors mRNA, but also increased the capacity of gingival fibroblasts to produce prostaglandin E2, a potent vasodilator agent^40^, in response to the kinins. Kinins themselves are also vasodilatory agents, and can be generated at the inflammatory site during periodontal infection by the action of gingipain, a kinin-producing protease expressed by *P. gingivalis*^41^. The proposed sequence of events involved in bacterial invasion promoted by *P. gingivalis*, including a role of TLR2 induced kinin expression in fibroblasts, is outlined in Fig. 7.

In conclusion, in this study we report that *P. gingivalis* LPS is able to up-regulate kinin receptors in human gingival fibroblasts and mouse gingiva by the activation of TLR2. Moreover, our data reveal a new pathway by which these receptors are up-regulated which is independent on the production of IL-1β and TNF-α in the case of B_1_ receptor, and partially dependent on TNF-α production in the case of B_2_ receptor. These findings open new horizons for studies investigating mechanisms controlling the expression of B_1_ and B_2_ receptors in non-inflammatory cells.

## Material and Methods

### Materials

Specified in Supplementary Material 1.

### Cell culture

Human gingival fibroblasts were isolated from healthy donors with written, informed consent as previously described^42^. Fibroblasts were from different individuals (males and females between 25–50 years of age (all generally and periodontally healthy) and the cells used in the present study were from passage 5–10. Approval from the Ethical Committee for Human Research at Umeå University was obtained for all the methods described, and all methods were performed in accordance with the relevant guidelines and regulations. The data shown were obtained using cells from one individual but reproduced using cells from another individual with the exception of the experiments used to produce data shown in Fig. 2 which were performed using cells from five different individuals.

### *In vivo* regulation of Bdkrb1 and Bdkrb2 by P. gingivalis LPS

In order to assess the effect of *P. gingivalis* LPS on the regulation of kinin receptors *in vivo*, we injected LPS from *P. gingivalis* (3 μg per injection), or PBS, in the gingiva in the mesial aspect of upper first molar of male 6-weeks old C57Bl/6 mice. The protocol for this experiment was approved by the Ethical Committee on Animal Experimentation at the School of Dentistry in Araraquara – UNESP, Brazil and performed in accordance with the guidelines from the Brazilian College for Animal Experimentation (COBEA). Injections were performed every second day for 14 days, and the animals were sacrificed 6 h after the last injection. The gingival tissue was dissected and the RNA was extracted using RNAqueous-MICRO kit for qPCR analysis.

### RNA extraction and cDNA synthesis

After exposure to the test substances for the time indicated in the graphs or figure legends, total RNA was extracted from the cells using RNAqueous-4PCR kit. The cDNA was synthesized with a first-strand cDNA synthesis kit using oligo(dT)_15_ as primers following the manufacturer’s instructions.

### Quantitative real-time polymerase chain reaction (qPCR)

The mRNA expression of human *BDKRB1*, *BDKRB2*, *TLR2*, *TLR4* and the mouse genes *Bdkrb1* and *Bdkrb2* were assessed using previously described primer sequences^3,27^. Amplification was performed in an ABI Prism 7900HT sequence detection system using cDNA as template, specific primers and probes and Taqman Universal Mater Mix kit. To control the amount of cDNA input, ribosomal protein L13A (*RPL13A*) or β-actin (*Actb*) were used as controls (housekeeping genes) for human and mouse samples, respectively.

### Radioligand binding assays

After overnight attachment of the fibroblasts, the media were changed and α-MEM with 1% FCS with or without Pam_2_CSK_4_ (50 ng/mL) was added. Twenty-four hours later, binding studies were performed following all the standardizations described previously^9^. To assess the amount of binding sites, the cells were incubated in MEM/HEPES/0.1% BSA with [^3^H]-BK 4 nmol/l or [^3^H]-des-Arg^10^-Lys-BK 14 nmol/l for 90 min at 4 °C. After extensive washing steps, the cells were detached and the radioactivity analyzed using liquid scintillation counter. The binding of [^3^H]-BK was competed for by B_2_, but not B_1_, ligands and the binding of [^3^H]-des-Arg^10^-Lys-BK was competed for by B_1_, but not B_2_, ligands (data not shown).

### Prostaglandin E_2_ production

The amount of PGE_2_ was measured in the supernatant of cells exposed to BK (1 μM) or DALBK (1 μM) for 24 hours by using a commercially available ELISA kit for PGE_2_. In order to analyze the effect of TLR2 activation on kinin receptors expression, cells were pre-treated with or without Pam_2_CSK_4_ (50 ng/mL) for 24 h prior to the addition of kinins.

### *TLR2* and *TLR4* knockdown

*TLR2* and *TLR4* were knocked down in gingival fibroblasts using siRNA as previously described^27^. Briefly, the cells were transfected with 30 nM of scrambled (SCR – Ambion, AM4635), TLR2 (Ambion, ID#111285) or TLR4 siRNA (Ambion, ID#112337) using lipofectamin 2000 in α-MEM with 10% FCS without antibiotics. The knockdown was confirmed by qPCR and more than 90% inhibition of *TLR2* and *TLR4* mRNA was achieved (data not shown). Twenty-four hours after transfection, the media were changed and the cells were exposed to the test substances; 6 h later RNA was extracted for qPCR analysis.

### Participation of IL-1β and TNF-α in up-regulation of kinin receptors induced by *P. gingivalis* LPS

After overnight attachment, human gingival fibroblasts were incubated in α-MEM/1% FCS in the presence or absence of *P. gingiavlis* LPS, with or without antibodies neutralizing human IL-1β or human TNF-α. The IL-1β neutralizing antibodies blocked IL-1β induced enhancement of *BDKRB1* mRNA expression and the TNF-α neutralizing antibodies blocked TNF-α induced increase of *BDKRB1* mRNA (Supplementary Material 2).

### Statistical analyses

Statistical analysis of multiple treatment groups was performed using analysis of variance (ANOVA), with Levene’s homogeneity test, and Dunnet’s T3 or Tukey post hoc test. For the experiments with two groups, the unpaired Student’s t-test was performed. The data shown in the figures are expressed as means ± standard error of means (SEM) for 3–6 wells per experimental group.

## Supplementary information

Supplementary 1

Supplementary 2

## Data Availability

The datasets generated during and/or analysed during the current study are available from the corresponding author on reasonable request.
