# Supplementary material for: Activation of Toll-like receptor 2 induces B1 and B2 kinin receptors in human gingival fibroblasts and in mouse gingiva
Source: Sci Rep. 2019 Feb 27;9:2973. doi: 10.1038/s41598-018-37777-z (PMC6393418; doi:10.1038/s41598-018-37777-z)
Supplement: Supplementary file 1 — Supplementary 1 [file 41598_2018_37777_MOESM1_ESM.pdf]

Activation of Toll-like receptor 2 induces B_1 and B_2 kinin receptors in human gingival fibroblasts and in mouse gingiva

Pedro P C Souza[1], *Pernilla Lundberg*[2], *Inger Lundgren*[2], *Fernando A Cintra-Magalhães*[1], *Claudio M Costa-Neto*[3], *Ulf H Lerner*[2,4,*]

1. Department of Physiology and Pathology, Sao Paulo State University (UNESP), School of Dentistry, Araraquara, SP, Brazil
2. Department of Molecular Periodontology, Umeå University, Umeå, Sweden
3. Department of Biochemistry and Immunology, Ribeirão Preto Medical School, University of São Paulo, Ribeirão Preto, SP, Brazil
4. Centre for Bone and Arthritis Research at Department of Internal Medicine and Clinical Nutrition, Institute for Medicine, Sahlgrenska Academy at University of Gothenburg, Gothenburg, Sweden

*Correspondence:
Professor Ulf H. Lerner, DDS, PhD
Centre for Bone and Arthritis Research at Department of Internal Medicine and Clinical Nutrition, Institute for Medicine, Sahlgrenska Academy at University of Gothenburg, Gothenburg, Sweden,
Mobile phone: +46 70 651 9103
E-mail: ulf.lerner@gu.se

Supplementary 1

Materials

The α-modification of minimal essential medium (α-MEM), fetal calf serum (FCS), RNAqueous-4PCR reagent kit, RNAqueous-MICRO kit, l-glutamine, oligonucleotide primers, fluorescence-labeled probes (reporter fluorescent dye VIC or FAM at the 5′ end and quencher fluorescent dye TAMRA at the the 3′ end), 384-well clear rxn plates, TaqMan Universal polymerase chain reaction (PCR) master mix, Lipofectamine 2000, and siRNAs were from Life Technologies (Carlsbad, CA); bradykinin (BK) and des-Arg^{10}-Lys-BK (DALBK) from Sigma (St. Louis, M.O.); [^{3}H]-des-Arg^{10}-Lys-BK and [^{3}H]-BK from DuMedical/NEN™ , Life Science Products (Boston, MA); *E. coli* LPS from Sigma (St. Louis, MO); *P. gingivalis* LPS (LPS-PG – Standard), and Pam_2CSK_4 from Invivogen (San Diego, CA); PGE_2 ELISA kit from Cayman Chemicals (Ann Arbor, MI); antibody neutralizing TNF-α from Hycult Biotech (Uden, NE); IL-1-β neutralizing antibody from R&D Systems (Abdington, UK); the multiwell plastic culture and petri dishes from Costar (Cambridge, MA) and the first-strand complementary DNA (cDNA) synthesis kit from Roche (Mannheim, Germany).
