# Supplementary material for: Activation of Toll-like receptor 2 induces B1 and B2 kinin receptors in human gingival fibroblasts and in mouse gingiva
Source: Sci Rep. 2019 Feb 27;9:2973. doi: 10.1038/s41598-018-37777-z (PMC6393418; doi:10.1038/s41598-018-37777-z)
Supplement: Supplementary file 2 — Supplementary 2 [file 41598_2018_37777_MOESM2_ESM.pdf]

Activation of Toll-like receptor 2 induces B_1 and B_2 kinin receptors in human gingival fibroblasts and in mouse gingiva

Pedro P C Souza[1], Pernilla Lundberg[2], Inger Lundgren[2], Fernando A Cintra-Magalhães[1], Claudio M Costa-Neto[3], Ulf H Lerner[2,4,]*

1. Department of Physiology and Pathology, Sao Paulo State University (UNESP), School of Dentistry, Araraquara, SP, Brazil
2. Department of Molecular Periodontology, Umeå University, Umeå, Sweden
3. Department of Biochemistry and Immunology, Ribeirão Preto Medical School, University of São Paulo, Ribeirão Preto, SP, Brazil
4. Centre for Bone and Arthritis Research at Department of Internal Medicine and Clinical Nutrition, Institute for Medicine, Sahlgrenska Academy at University of Gothenburg, Gothenburg, Sweden

*Correspondence:
Professor Ulf H. Lerner, DDS, PhD
Centre for Bone and Arthritis Research at Department of Internal Medicine and Clinical Nutrition, Institute for Medicine, Sahlgrenska Academy at University of Gothenburg, Gothenburg, Sweden,
Mobile phone: +46 70 651 9103
E-mail: ulf.lerner@gu.se

Supplementary 2

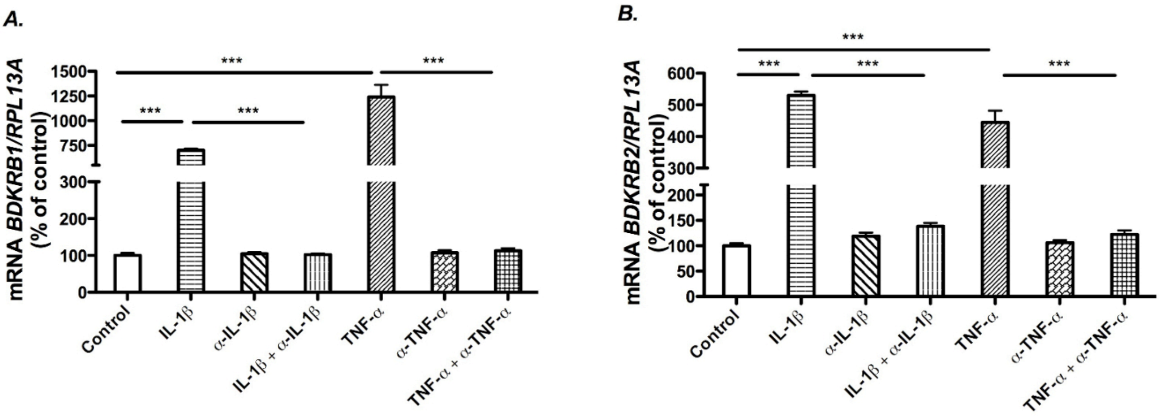

Supplementary 2 – The capacity of specific blocking antibodies to impair the effects of IL-1β and TNF-α. Gingival fibroblasts were exposed to IL-1β (100ng/mL) and TNF- α (50μg/mL) in the presence or absence of anti-IL-1β (0.3 μg/mL) or anti-TNF-α 1 (μg/mL), respectively, and the expression of *BDKRB1* (***A***) and *BDKRB2* (***B***) mRNA was analyzed by qPCR using Taqman Assays. Data were normalizad against *RPL13A* and expressed as percent of control that was arbitrarily set to 100%. Data are expressed as means ±SEM (n=4, ***$p<0.001$).
